# Oxygenase Ppo-Regulated Moldy Volatiles Affect Growth, Pathogenicity and Patulin Biosynthesis of *Penicillium expansum* Through G Protein Signaling

**DOI:** 10.3390/jof10120827

**Published:** 2024-11-27

**Authors:** Di Gong, Tingting Yan, Xuexue Wang, Dov Prusky, Danfeng Long, Ying Zhang, Yang Bi

**Affiliations:** 1 School of Public Health, Lanzhou University, Lanzhou 730000, China; yantt2023@lzu.edu.cn (T.Y.); longdf@lzu.edu.cn (D.L.); yingz@lzu.edu.cn (Y.Z.); 2 College of Food Science and Engineering, Gansu Agricultural University, Lanzhou 730070, China; wxx4121@163.com; 3 Department of Postharvest and Food Science, Agricultural Research Organization, Volcani Center, Rishon LeZion 7505101, Israel; dovprusk@volcani.agri.gov.il

**Keywords:** *Penicillium expansum*, psi-producing oxygenase, C8 moldy VOCs, G protein signal transduction, patulin biosynthesis, pathogenicity

## Abstract

Precocious sexual inducer (psi)-producing oxygenases (Ppos) participate in the production of C8 moldy volatile compounds (MVOCs), and these compounds could act as signal molecules modulating G protein signaling cascades, which participates in the growth and development, secondary metabolisms and pathogenicity of filamentous fungi. In this study, PePpoA and PePpoC proteins were identified in *Penicillium expansum*. The deletion of *ppoA* decreased C8 MVOC production in *P. expansum*, while they were not detected in the *ΔppoC* strain (*p* < 0.05). In addition, down-regulated cAMP/PKA and PKC/PLC signaling showed in the two mutants (*p* < 0.05). The two mutants showed slow colony growth and down-regulated expression of genes regulating spore development (*abaA*, *wetA*, *brlA* and *vosA*) with broken morphology of spore and hyphae. In addition, the two mutants had decreased pathogenicity on apple fruit and less patulin production in vitro and in vivo. Compared with *ΔppoA* strain, the deletion of *ppoC* inhibited G protein signaling pathways more, and the *ΔppoC* strain had more defective growth and development as well as reduced pathogenicity and patulin production (*p* < 0.05). Therefore, PePpoC proteins affect more growth and development, patulin biosynthesis and pathogenicity of *P. expansum* by regulating C8 MVOC-mediated G protein signaling transduction.

## 1. Introduction

A moldy odor, described as a ‘mushroom’ or ‘earthy’ note, is considered as a characteristic odor that is mainly released by fungi [1]. Various fungi have been proved to produce these odors, including *Aspergillus* genus, *Penicillium* genus, *Alternaria* genus and *Fusarium* genus [2]. The moldy odor produced by fungi infecting fruits significantly reduces fruit quality and has been associated with allergic reactions in humans [3]. C8 moldy volatile compounds (MVOCs), including 1-octen-3-ol, 3-octanol, 3-octanone and (E)-2-octen-1-ol, are the main compositions of moldy odor and they could affect growth, development, pathogenicity and mycotoxin production of fungi [4]. Treatment with 1-octen-3-ol inhibited the colony diameter, sclerotia formation and spore density of *Aspergillus flavus* [5]. Moreover, 1-octen-3-ol inhibited colony growth, sporulation and virulence-associated gene expression of *Botrytis cinerea* [6]. In addition, 3-octanol controls gray mold of grapes by inducing autophagy, suppressing conidial germination and reducing cell viability of *B. cinerea* [7]. It also inhibited aflatoxin production in *A. flavus* at the metabolic level, but enhanced patulin production in *P. expansum* incubated on patulin-suppressing medium by regulating genes expression involved patulin biosynthesis [5,8].

Precocious sexual inducer (psi)-producing oxygenases (Ppos) are a class of cyclooxygenase-like enzymes, which can directly regulate the biosynthesis of C8 MVOCs in fungi [2]. Ppo proteins are conserved in filamentous fungi and have four sub-types, including PpoA, B, C or D, which behave differently in the position of oxygenation of unsaturated fatty acids [9]. Polyunsaturated fatty acids such as oleic acid, linoleic acid and linolenic acid can be oxidatively cleaved to various oxylipins by Ppo proteins. And these oxylipins such as psi factor participate in the growth, development and secondary metabolisms of fungi [10]. 1-octen-3-ol was only not detected in the *ppoC* knocked out strain of *A. luchuensi* [11]. Moreover, loss of *ppoC* inhibited 1-octen-3-ol production in *Podospora anserina* [12]. Except regulating C8 MVOC production, Ppo proteins also participate in the regulation of fungal growth, development, pathogenicity and mycotoxin production [13,14,15]. Deletion of *ppoA*, *B* or *C* significantly down-regulated the expression of *brlA*, a transcription factor that regulates fungal spore production, and reduced sexual spore production of *A. nidulans* [14]. In *A. fumigatus*, loss of *ppoA* or *ppoC* has little effect on the phenotype, but knocking out *ppoC* reduced spore production, altered conidial shape, improved tolerance to oxidative stress and increased uptake and killing of the fungus by primary alveolar macrophages [16]. In addition, the deletion of *ppoA*, *B*, *C* or *D* decreased cell density development, reduced fungal colonization of peanut seed and inhibited aflatoxin production in *A. flavus* [17].

C8 MVOCs are classified as oxylipin, which can act as a signal molecule and enter into cells via specific transporters, and then bind to host G-protein-coupled receptor (GPCRs), activating downstream signaling cascades [18]. G protein signaling pathways are a ubiquitous transmembrane signaling pathway in filamentous fungi, which is involved in recognizing and transmitting multiple extracellular signaling stimuli that have a major impact on the fungal growth, development, pathogenicity and secondary metabolisms [19]. *P. expansum* is an important pathogenic fungi that not only causes blue mold in various fruits, but also releases C8 MVOCs and produces mycotoxins, such as patulin, posing a risk to human and animal health [20]. Therefore, we hypothesize that PePpoA and PePpoC regulate growth, pathogenicity and mycotoxin production through C8 MVOC-mediated G protein signaling in *P. expansum*. However, little study focuses on C8 MVOC biosynthesis and their functions in *P. expansum*. Therefore, the aims of this study were to identify Ppo proteins in *P. expansum*, and then to construct the *ppoA* and *ppoC* deletion strains of *P. expansum* to (1) determine the C8 MVOC production of the strains; (2) observe the growth, development and pathogenicity of the strains; and (3) analyze patulin accumulation and expression of patulin biosynthetic cluster genes of the strains.

## 2. Materials and Methods

### 2.1. Fungal Strains and Culture Conditions

*P. expansum* T01 strain was obtained from Prof. Shiping Tian, Institute of Botany, Chinese Academy of Sciences, China. The genomic characterization and patulin biosynthesis of the strain have been clarified by Prof. Tian’s team [21]. This wild type (WT) strain was used to construct all the mutants in the experiment. All the strains were cultured at 25 °C in the dark on PDA medium. After 7-day incubation, the conidia of each strain were harvested using 5 mL of sterile distilled water. The concentration of spore suspension for each strain was adjusted using a hemocytometer (BioSharp, Hefei, China).

### 2.2. Bioinformatic Analysis

NCBI (https://www.ncbi.nlm.nih.gov/, accessed on 13 June 2023) was used to blast the gene sequence of *ppoA* (AYHP01000411.1) and *ppoC* (AYHP01000475.1) in *P. expansum* T01 based on the gene and amino acid sequence of them in *P. expansum* MD-8. The E-value threshold was set to 0.05, the Word size was set to 28 and the +1/−2 (match +1, mismatch −2) matrix was used for comparison. The conserved domains of PpoA and PpoC in *P. expansum* T01 were assayed by Conserved Domains of NCBI, and the multiple sequence comparison was performed by DNAMAN. The phylogenetic trees of PpoA and PpoC of *P. expansum* T01 were constructed by the Neighbor-joining method using MEGA11 software v10.2 and the Bootstrap method was set to 1000.

### 2.3. Construction of Knockout and Complementary Strains

According to the description by Zhang et al. [22], the deletion vectors of *ppoA* and *ppoC* were generated using a hygromycin B resistance marker. The *ppoA* and *ppoC* genes’ genomic flanking regions were PCR amplified using specific primer pairs and then mixed with the predigested pCHPH vector to obtain the deletion vectors. The corresponding complementary vectors were generated using a neomycin resistance marker. For each gene-complementary plasmid, the genomic regions of each target gene was amplified using specific primer pairs and then mixed with the predigested the pCNEO vector to obtain the complementary vectors. Subsequently, the vectors were used for chemical transformation of high-efficiency *Escherichia coli* DH5αcells (AngYuBio, Shanghai, China). Kanamycin-resistant transformants were screened by PCR. And then, the plasmid was transformed into *Agrobacterium tumefaciens* EHA105 and then co-cultured with the WT or the corresponding knockout mutants to obtain the knockout (*ΔppoA* and *ΔppoC*) or their complementary (*ΔppoA-C* and *ΔppoC-C*) strains. Hygromycin-resistant colonies were confirmed by PCR and RT-qPCR analysis. In total, 17 and 19 colonies were collected for the verification of *ppoA* and *ppoC* knocking out strains and the success rate of conversion was 57.14% and 77.78%, respectively. All primers used to construct and verify the mutant strains are listed in Table S1. The results of PCR verification and RT-qPCR quantification are shown in Figures S1 and S2.

### 2.4. C8 MVOC Production

A 2-µL spore suspension containing 1 × 10^6^ spores mL^−^^1^ of either the WT and the mutants was inoculated on PDA media, and then incubated at 25 °C in the dark. After a 7-day incubation, 5 g of mycelia and spores with PDA medium from each strain were collected into a 20 mL headspace vial. The vials were equilibrated in a laboratory stirrer/hot plate (ZNCL-BS, EXCEED, Qingdao, China) at 40 °C for 30 min and C8 VOCs were extracted using 50/30 μ mpolydimethylsiloxane/divinylbenzene/carboxen fiber (PDMS/DVB/CAR) (Supelco, Inc., Bellefonte, PA, USA). After extraction, the SPME device was inserted into GC-MS (8890 5597C, Agilent Technologies Co., Ltd., Santa Clara, CA, USA) equipped with a DB-WAX column (60 m × 0.32 mm with a 1 mm film thickness) (Agilent Technologies Co., Ltd., USA) to quantify the release of C8 VOCs. The injection port temperature was 220 °C and 1 mL min^−^^1^ of helium was used as the carrier gas. The GC oven temperature was held at 40 °C for 3 min, increased by 5 °C min^−^^1^ to 150 °C, 10 °C min^−1^ to 220 °C and then held for 5 min. Mass spectra were obtained by electron ionization at 70 eV with a scanning range of 20–500 mass units. Compounds were identified by comparing the spectra with the NIST-98/Wiley library and matching retention index of authentic reference standards. A quantitative determination of VOCs was calculated based on relative peak area % [23]. The uninoculated media was used as a blank.

### 2.5. Observation of Colony Growth and Sporulation

A 2-µL spore suspension containing 1 × 10^6^ spores mL^−^^1^ of either the WT and the mutants was inoculated onto PDA media and then incubated at 25 °C in the dark. Colony growth was photographed and determined by diameter measurement every 2 d up to 8 d using three replicate plates per strain. Spores of each strain were obtained by adding 5 mL of sterile water to the plate and sporulation was counted using a hemocytometer [24].

Mycelium and spores from each strain cultured on PDB liquid medium and PDA plates separately were attached to copper sheets to observe the morphology by scanning electron microscopy (SEM) (SEM3200A, Chinainstru & Quantumtech (Hefei) Co., Hefei, China) [25]. Samples with 1 mL of 2.5% glutaraldehyde solution were gently shaken and then stored at 4 °C overnight. After that, the samples were dehydrated with ethanol solution by gradient concentration (25%, 50%, 70%, 95% and 100%) for 10 min. The samples were centrifuged at 5000 rpm for 3 min and then air dried for the observation.

### 2.6. Pathogenicity Assay

Apple fruits, with uniform size and commercial maturity, without wounds and injury, were selected and were washed with tap water and then soaked in 0.1% sodium hypochlorite for 2 min. After air drying, artificial holes were made at equatorial parts of the fruits. Each hole was inoculated with 10 μL 1 × 10^6^ spores mL^−^^1^ of each strain and incubated for 8 d at room temperature. The decay symptoms and lesion diameter on fruit was recorded every 2 d of incubation [22]. Three fruits were used for each strain and replicated three times.

### 2.7. Patulin Production

A 2-µL spore suspension containing 1 × 10^6^ spores mL^−^^1^ of either the WT and the mutants was inoculated on PDA media and then incubated at 25 °C in the dark. After a 7-day incubation, spores were washed with 5 mL of sterile water, and then centrifuged to collect the supernatant. The supernatant was filtered through a 0.45 µm filter membrane (Agela Technologies, Tianjin, China) and then kept at −20 °C for patulin analysis. In the in vivo experiment, apple fruits were inoculated with 1 × 10^6^ spores mL^−^^1^ of each strain and incubated for 7 d at room temperature. The decayed tissues were taken and crushed in 25 mL of distilled water and then shaken at 150 rpm for 1 h. After that, the supernatants were collected and extracted with ethyl acetate three times and then the extracts were dried with a rotary evaporator and dissolved in sterile water (2 mL, pH 4.0). The mixture was filtered through a 0.45 µm filter membrane (Agela Technologies, Tianjin, China) and then kept at −20 °C for patulin analysis [22,26].

High-performance liquid chromatography (HPLC, 1260, Agilent Technologies Co. Ltd., USA) analysis was used to analyze patulin production. Patulin production was quantitatively analyzed by injecting 10 µL into an HPLC system equipped with a C18 reverse-phase column (250 nm × 4.6 nm) and an UV absorption detector [27,28]. The mobile phase was acetonitrile: water (10:90, V:V) at a flow rate of 1 mL min^−^^1^. The quantification of patulin content was carried out by comparison with a calibration curve of the standard mycotoxin (Sigma, Darmstadt, Germany), which was freshly prepared before analysis. The results were expressed as μg mL^−^^1^.

### 2.8. RNA Isolation and RT-qPCR Analysis of Gene Expression

Mycelia grown on PDA medium were harvested on the 5th day of incubation and then frozen in liquid nitrogen immediately and kept at −80 °C for use. Total RNA was extracted from 100 mg of the samples using TRNzol Reagent (TIANGEN, Beijing, China) based on the manufacturer’s protocol and the cDNA Synthesis Kit (Accurate Biology, Changsha, China) was used to synthesize cDNA. The RT-qPCR was performed using the SYBR^®^ Green Premix Pro qPCR Kit (Accurate Biology, China) in the ABI StepOnePlus Real-Time PCR System (Applied Biosystems, Carlsbad, CA, USA). The samples were normalized using *β-tubulin* as the endogenous control and the relative expression levels were measured using the 2(^−ΔΔCt^) analysis method [29]. Melt curve analysis was also performed to enhance specificity. The primers used for the RT-qPCR analysis are listed in Table S2.

### 2.9. Statistical Analysis

All experiments described here were repeated at least three times as independent experiments. The data were analyzed by SPSS 26.0 (SPSS Inc., Chicago, IL, USA) and the figures were drawn by GraphPad Prism v9.5. Multiple comparisons were performed by one-way analysis of variance (ANOVA) and the significant difference was analyzed by Duncan’s multiple comparison, with *p* < 0.05 indicating significant differences.

## 3. Results

### 3.1. Bioinformatic Analysis of PePpoA and PePpoC

**Figure 1:**
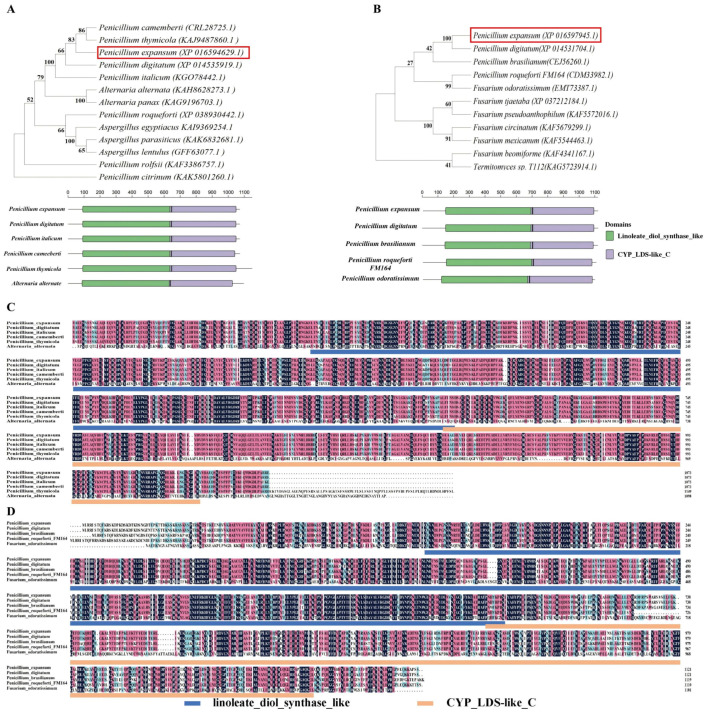
Phylogenetic tree and conserved structural domains of PePpoA and PePpoC protein with other fungal PpoA (**A**) and PpoC (**B**). The multiple sequence comparison of PePpoA (**C**) and PePpoC (**D**) protein with other fungi. The number of Bootstrap iterations is 1000, which increases the node support value and improves the reliability of homology.

The PpoA and PpoC proteins in *P. expansum* T01 contained 1073 and 1121 amino acids, respectively. Phylogenetic analyses showed that the PpoA protein in *P. expansum* had high homology with the PpoA protein in *P. camemberti* (83%) and *P. digitatum* (66%), and it had low homology with the PpoA protein in *P. roqueforti* (40%) (Figure 1A). The PpoC protein in *P. expansum* had high homology with the PpoC protein in *P. digitatum* (100%) and it had low homology with the PpoC protein in *P. brasilianum* (42%) and *Fuarium odortissimum* (27%) (Figure 1B). Both of the PePpoA and PePpoC proteins contain two highly conserved structural domains, including linoleate_diol_synthase_like and CYP_LDS-like_C (Figure 1C,D).

### 3.2. PePpoC Is Required for C8 MVOC Production in P. expansum

**Figure 2:**
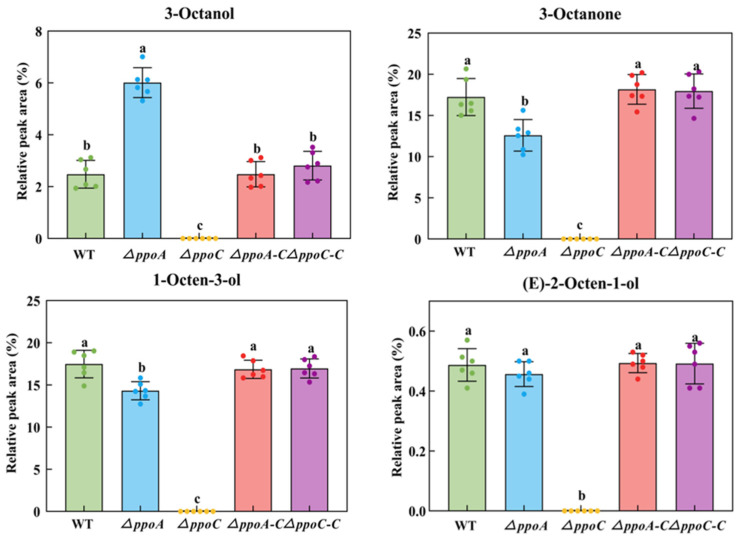
Production of C8 MVOCs in the *ΔppoA* and *ΔppoC* strain. Bars indicate the standard error. Different letters indicate significant differences in different groups (*p* < 0.05).

C8 MVOCs, including 1-octen-3-ol, 3-octanol, 3-octanone and (E)-2-octen-1-ol, are considered to be characteristic compounds released by fungi, which contribute to moldy odor [2]. Compared with the WT strain, the deletion of *ppoA* enhanced the production of 3-octanol by 1.43-fold, but inhibited the production of 3-octanone and 1-octen-3-ol by 26.99% and 18.03% in *P. expansum*, respectively (*p* < 0.05) (Figure 2). There was no significant difference in the production of (E)-2-octen-1-ol between the WT and *ΔppoA* strain. However, the production of 1-octen-3-ol, 3-octanol, 3-octanone and (E)-2-octen-1-ol was not detected in the *ΔppoC* strain. The complementary strains restored the production of these C8 MVOCs as in the WT. These results indicate that PePpoA protein could affect the production of C8 MVOCs, but PePpoC protein is the key to regulating the production of these MVOCs in *P. expansum*.

### 3.3. Loss of PePpoA and PePpoC Down-Regulated the Gene Expression Involved in cAMP-PKA and PLC/PKC Signaling Pathways in P. expansum

**Figure 3:**
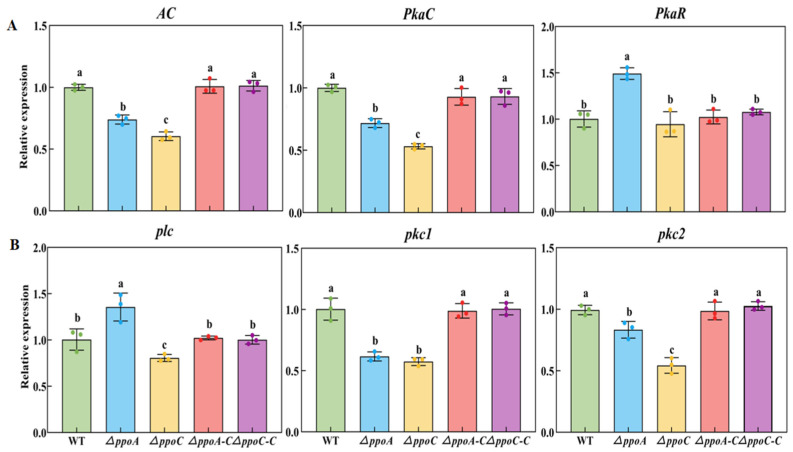
Gene expression involved in the cAMP/PKA (**A**) and PLC (**B**) signaling pathway in the *ΔppoA* and *ΔppoC* strain. Bars indicate the standard error. Different letters indicate significant differences in different groups (*p* < 0.05).

*AC*, *PkaC* and *PkaR* are important genes participating in the cAMP-PKA signaling pathway in fungi [20]. Deletion of *ppoA* or *ppoC* down-regulated the gene expression of *AC* and *PkaC*, while the *ΔppoC* strain had lower expression levels (Figure 3A). Compared with the WT strain, the expression levels of *AC* and *PkaC* were 39.57% and 28.25%, and 26.06% and 46.83% lower in the *ΔppoA* and *ΔppoC* strains, respectively. However, the deletion of *ppoA* increased the expression of *PkaR*, which was 49.23% higher than that of the WT strain. *plc*, *pkc1* and *pkc2* are important genes participating in the PLC/PKC signaling pathway in fungi [20]. Deletion of *ppoA* and *ppoC* down-regulated the expression of *pkc1* and *pkc2* (Figure 3B). Compared with the WT strain, the expression levels of *pkc1* and *pkc2* were 38.37% and 16.68%, and 42.57% and 45.70% lower in the *ΔppoA* and *ΔppoC* strains, respectively. However, deletion of *ppoA* increased the expression of *plc*, which was 35.60% higher than that in the WT strain. These results indicated that deletion of either *ppoA* or *ppoC* down-regulated the gene expression involved in the cAMP-PKA and PLC/PKC signaling pathways in *P. expansum*.

### 3.4. Loss of PePpoA or PePpoC Affects Colony Growth and Hyphal Morphology of P. expansum

**Figure 4:**
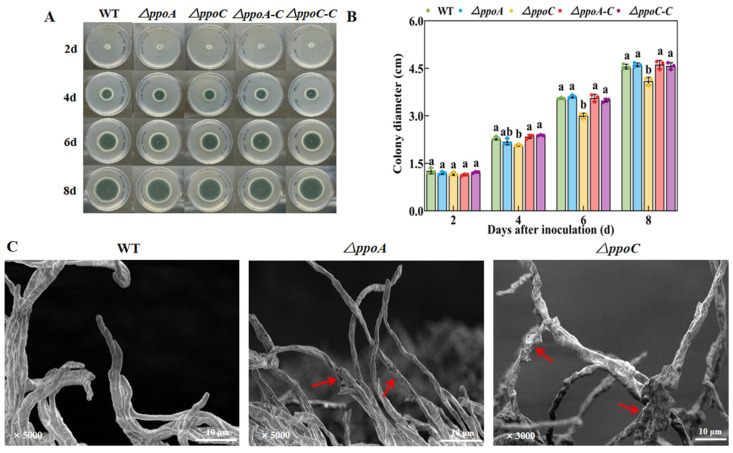
Pictures of radial growth (**A**), colony diameter (**B**) and hyphal morphology (**C**) in the *ΔppoA* and *ΔppoC* strains. Bars indicate the standard error. Different letters indicate significant differences in different groups at the same time (*p* < 0.05). Red arrows indicate changes in hyphal morphology.

The *ΔppoA* and *ΔppoC* strains had smaller colony diameters compared with the WT and their corresponding complementary strains after 4 d of incubation, while the *ΔppoC* strain had a smaller colony diameter compared with the *ΔppoA* strains (Figure 4A,B). The colony diameter of the *ΔppoC* strain was 12.76% and 10.48% lower than than that of the WT and *ΔppoA* strain on the 8th day of incubation, respectively (*p* < 0.05) (Figure 4B). The results of SEM showed that the deletion of *ppoA* or *ppoC* resulted in a dried out, ruffled and broken hyphal morphology in *P. expansum* compared with the WT strain. Moreover, more thick and hyphal branches were found in the *ΔppoC* strain compared with the WT and *ΔppoA* strain (Figure 4C). These results indicate that PePpoC protein shows more effects on the colony growth and hyphal development in *P. expansum* compared with PePpoA protein.

### 3.5. Loss of PePpoA or PePpoC Affects Sporulation and Spore Morphology of P. expansum

**Figure 5:**
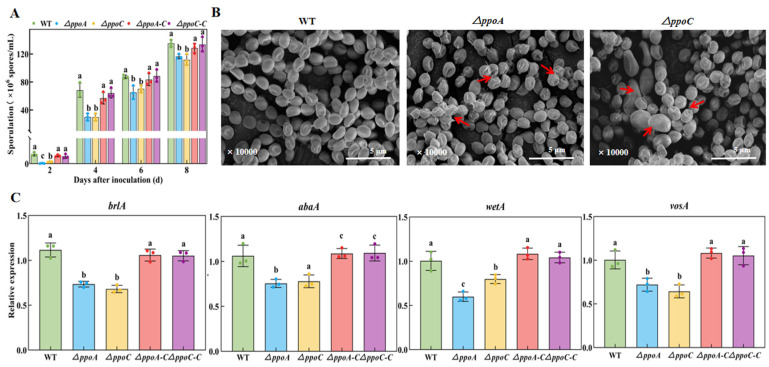
Sporulation (**A**), spore morphology (**B**), and sporulation-related genes expression (**C**) in the *ΔppoA* and *ΔppoC* strains. Bars indicate the standard error. Different letters indicate significant differences in different groups (*p* < 0.05). Red arrows indicate changes in spore morphology.

The sporulation decreased in the *ΔppoA* and *ΔppoC* strains compared with the WT and their complementary strains, while there is no significant difference between the two mutants (Figure 5A). On the 4th day of incubation, the sporulation of the *ΔppoA* and *ΔppoC* strains was only 44.12% and 44.10% of the WT strain, respectively (*p* < 0.05). Compared with the WT and the corresponding complementary strains, the spore surface of the *ΔppoA* strain was wrinkled, sunken and had a large number of burrs, whereas the spore surface of the *ΔppoC* strain was wrinkled, sunken and had a few burrs, and some of the spores were enlarged in the *ΔppoC* strain (Figure 5B).

*brlA*, *abaA*, *wetA* and *vosA* are critical genes regulating spore formation and development in fungi [29]. The expression of these genes was lower in both of the two mutants compared with the WT and the their complementary strains (Figure 5C). Compared with the WT strain, the expression of *brlA*, *abaA*, *wetA* and *vosA* in the *ΔppoA* strain was decreased by 34.23%, 24.50%, 40.20% and 28.10%, which was decreased by 38.74%, 22.20%, 20.20% and 32.80% in the *ΔppoC* strain, respectively (*p* < 0.05). Notably, the expression of *wetA* was 25.06% lower in the *ΔppoA* strain than that in the *ΔppoC* strain (*p* < 0.05). These results indicate that the deletion of *ppoA* or *ppoC* decreases sporulation and causes abnormal spore morphology in *P. expansum*.

### 3.6. Loss of PePpoA or PePpoC Decreases Pathogenicity of P. expansum on Apple Fruit

**Figure 6:**
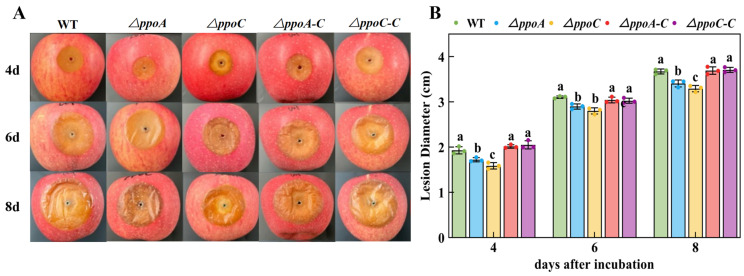
Decay symptoms (**A**) and lesion diameter (**B**) on apple fruits inoculated with *ΔppoA* and *ΔppoC* strains. Bars indicate the standard error. Different letters indicate significant differences in different groups (*p* < 0.05).

During the incubation, darker decay spots present on apple fruit inoculated with *ΔppoC* strain compared with other strain’s inoculation (Figure 6A). The deletion of either *ppoA* or *ppoC* decreased pathogenicity of *P. expansum* on apple fruit, while smaller lesion diameter found in the *ΔppoC*-inoculated fruit (Figure 6). Especially on the 4th day of incubation, the lesion diameter of fruit caused by *ΔppoC* strian inoculation was 17.73% lower compared with the WT strain inoculation (*p* < 0.05).

### 3.7. Loss of PePpoA or PePpoC Inhibits Patulin Biosynthesis in P. expansum

**Figure 7:**
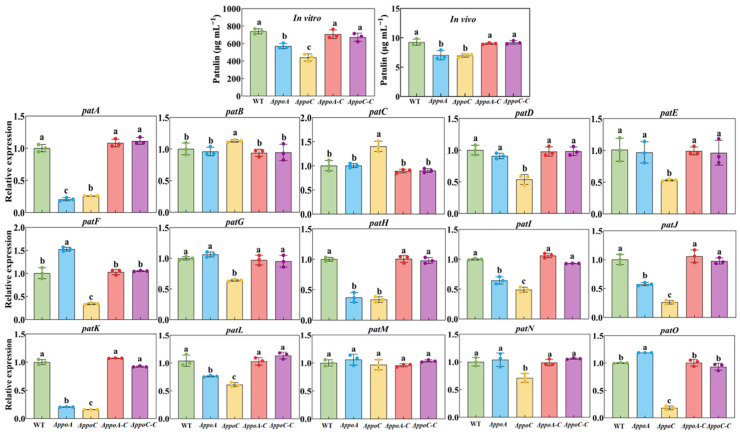
The patulin production of strains in vitro and in vivo, and expression levels of patulin biosynthetic cluster genes in the *ΔppoA* and *ΔppoC* strain. Bars indicate the standard error. Different letters indicate significant differences in different groups (*p* < 0.05).

In vitro, less patulin was accumulated in the *ΔppoA* and *ΔppoC* strains, which decreased by 22.84% and 40.41% compared with the WT strain, respectively (Figure 7). Moreover, compared with the *ΔppoA* strain, the patulin production was 22.77% lower in the *ΔppoC* strain (*p* < 0.05). In vivo, less patulin accumulated in the decay tissues caused by *ΔppoA* and *ΔppoC* strain, which decreased by 24. 02% and 24.68% compared with the WT strain (*p* < 0.05) (Figure 7). The expression levels of the most genes involved in the patulin biosynthetic gene cluster were down-regulated in the *ΔppoA* strain (Figure 7). For example, *patA*, *patH*, *patI*, *patJ*, *patK* and *patL* were expressed at 79.42%, 62.81%, 35.50%, 42.40%, 79.57% and 25.10% lower than that in the WT strain (*p* < 0.05). Except *patB*, *patC* and *patM*, the expression levels of other patulin biosynthesis-related genes were significantly down-regulated in the *ΔppoA* strain (*p* < 0.05). Compared with the WT strain, the expression levels of *patA*, *patD*, *patE*, *patF*, *patG*, *patH*, *patI*, *patJ*, *patK*, *patL*, *patN* and *patO* were 74.22%, 46.32%, 46.94%, 65.65%, 35.97%, 66.11%, 51.02%, 73.74%, 83.92%, 38.61%, 28.84% and 81.90% lower than that in the WT strain (*p* < 0.05). Notably, compared with the *ΔppoA* strain, lower expression levels of patulin biosynthesis genes were found in the *ΔppoC* strain. The complementary strains restored the patulin biosynthesis as in the WT strain. These results indicate that the deletion of *ppoA* or *ppoC* inhibits patulin biosynthesis in *P. expansum*, whereas *ppoC* deletion leads to a greater impact.

## 4. Discussion

In the present study, two Ppo proteins, PpoA and PpoC, were identified in the *P. expansum* T01 strain. The conserved structural domains of both PePpoA and PePpoC proteins have a high homology with those of *P. digitatum* (Figure 1A,B), suggesting that the PePpoA and PePpoC proteins in *P. expansum* T01 might be homologous to the PpoA and PpoC proteins in *P. digitatum*. We hypothesized that the PpoA and PpoC proteins in *P. expansum* had a high homology with these proteins in *P. digitatum*, which may be due to them belonging to the *Penicillium* genus and having similar fruit hosts. In addition, the two fungi may show small differences in ITS sequences, supporting their similar genetic background at the genomic level. In addition, both of the PePpoA and PePpoC proteins contained two highly conserved domains, including linoleate_diol_synthase_like and CYP_LDS-like_C (Figure 1C,D). The linoleate_diol_synthase_like structural domain catalyzes the reaction of dioxygenase with unsaturated fatty acids, whereas the CYP_LDS-like_C structural domain catalyzes the rearrangement of fatty acid hydroperoxides formed from the linoleate_diol_synthase_like structural domain [9].

C8 MVOCs belong to oxylipins and are considered as the main compositions of moldy odor released by fungi [2]. These C8 MVOCs released by fungi are mainly derived from the oxidative cleavage of linoleic acid via the Ppo pathway. In fungi, the oxidation of linoleic acid by Ppo produces 10-hydroperoxide (10-HPOD), which is subsequently non-enzymatically cleaved to 1-octen-3-ol and 10-oxodecanoic acid (10-ODA) [30]. Although several Ppo proteins exist in filamentous fungi, not all of them regulate the biosynthesis of C8 MVOC. It has been reported that four genes encoding Ppo proteins have been identified in the *Aspergillus* genus, including *ppoA*, *ppoB*, *ppoC* and *ppoD*; among them, *ppoC* gene encoding a 10-dioxygenase that catalyzes the breakdown of linoleic acid to generate 10-HPOD, regulating the synthesis of 1-octen-3-ol, 2-octen-1-ol, 2-octenal and 3-octanone [10,17]. The PpoA protein existing in *Aspergillus* species convert linoleic acid into 8-HODE and 5,8-diHODE [31]. In the study, the deletion of *ppoA* affected the production of 3-octanol, 1-octen-3-ol, 3-octanone and (E)-2-octen-1-ol in *P. expansum*, while all of these VOCs were not detected in the *ppoC* knocked out mutant (Figure 2). These results are similar with the results that when deleting *ppoA*, *ppoC* or *ppoD* in *A. luchuensis*, only 1-octent-3-ol was not detected in the *ppoC* knocked out mutant [32]. In addition, rice koji inoculated with *A. luchuensis* deleted *ppoA* produced the similar 1-octen-3-ol compared with that inoculated with the WT strain, whereas 1-octen-3-ol was not detected in rice koji inoculated with the *ppoC* knocked out strain [11]. The loss of *ppoC* directly inhibited 1-octen-3-ol production in *Podospora anserina* [12]. Notably, the deletion of *ppoA* up regulated the expression of *ppoC* (Figure S2), which may contribute to the production of C8 MVOCs in the *ΔppoA* strain. Therefore, we believe that PePpoA protein could affect the production of C8 MVOCs, but PePpoC protein is the key to regulate the production of C8 MVOCs in *P. expansum*.

C8 volatile oxylipins participate in signal transduction in fungi [33]. As quorum-sensing molecules, these compounds have functions as hormone-like signals that could active G protein signaling pathways in filamentous fungi [10]. These signals are received by GPCRs, and then actives signal transmission of G protein to adenylate cyclase, which could initiate downstream cAMP-PKA pathway by regulating intracellular cAMP levels [34,35]. Moreover, G protein could promote PLC to catalyze hydrolysis of phosphatidylinositol 4,5-bisphosphate, producing inositol 1,4,5-trisphosphate and diacylglycerol, which induces the release of intracellular Ca^2+^ and activate the PLC/PKC signaling pathway [36,37]. Our study found that the deletion of either *ppoA* or *ppoC* down-regulated the genes expression involved in cAMP-PKA and PLC/PKC signaling pathways in *P. expansum*, while *ppoC* knocked out showed stronger effects (Figure 3). It has been reported that exogenous oxylipins stimulated a burst in cAMP under the mediation of GPCR GprD [38]. Since the release of C8 oxylipins were inhibited in the *ΔppoA* and *ΔppoC* strains (Figure 2), we hypothesized that the deletion of *ppoA* or *ppoC* affect the cAMP-PKA and PLC/PKC signaling cascades in *P. expansum* may by inhibiting C8 MVOC production, resulting in the inability to activate the GPCRs that bind to it.

G protein signaling pathways respond to oxylipin signals, participating in the regulation of fungal growth and development by activating the expression levels of developmental regulated genes [39]. In the study, the deletion of either *ppoA* and *ppoC* decreased colony growth, caused deficient morphology of hyphae and spores, reduced sporulation and down-regulated expression levels of the transcription factors *brlA*, *abaA*, *wetA* and *vosA* associated with spore development of *P. expansum* (Figure 4 and Figure 5). Brown et al. [17] illustrated that the deletion of *ppoA*, *ppoC* or *ppoD* inhibited sporulation of *A. flavus*. The growth and morphogenesis of fungal mycelium depends to a large extent on the processes of vesicle transport and cytoskeletal dynamics. During this process, Golgi-derived secretory vesicles accumulate at the tip of the mycelium to mediate membrane fusion and participate in mycelial branching and apical extension by secreting their contents [40]. G protein signaling pathways regulate the plasma membrane signaling pathway and the cytoskeleton influencing fungal growth and development [41]. In addition, the loss of *ppoA*, *ppoB* and *ppoC* down-regulated the expression of *brlA* in *A. nidulans*, a transcription factor that regulates fungal sporulation [14]. Hyphae growth and spore development are regulated by the cAMP-PKA signaling pathway in filamentous fungi, which could respond to cell signals, and activates the expression of hyphal-specific factors and development-regulated genes, thus participating in the regulation of fungal growth and development [42]. The deletion of *ppoA* or *ppoC* inhibited C8 MVOC production that may reduce the responses of the cAMP-PKA signaling pathway to C8 oxylipin signals, thereby limiting the growth and development of *P. expansum*. In addition, the deletion of *ppoA* or *ppoC* decreased pathogenicity of *P. expansum* on apple fruit (Figure 6), which may be related to the morphological defects in hyphae and spores affecting their normal functions. The *ppoC* gene had more effects on the growth and development of *P. expansum*, which may cause less pathogenicity of *ΔppoC* to apple fruit compared with the *ΔppoA* strain. In addition, it has been proved that C8 MVOCs, especially 1-octen-3-ol, could inhibit fungal growth and development in vitro and in vivo, including *Aspergillus*, *Fusarium*, *Penicillium* and *Alternaria* species [2]. We hypothesized that the reduction of pathogenicity in *ΔppoC* strain may related to the inhibition of 1-octen-3-ol production, which may directly regulate pathogenicity of *P. expansum*.

Patulin is an important mycotoxin produced by *P. expansum* and it could cause hepatotoxicity and genotoxicity, posing a threat to human health [43]. In the study, the deletion of either *ppoA* or *ppoC* reduced patulin production in vitro and in vivo (Figure 7). A total of 15 genes (*patA-patO*) are arranged in the patulin biosynthetic gene cluster, including 1 transcription factor (*patL*), 3 transporter proteins (*patA*, *patC* and *patM*) and 11 biosynthetic enzymes. The expression levels of most of these genes were down-regulated in the two mutants (Figure 7). These results are consistent with that of the deletion of *ppo* in *Fusarium sporotrichioides* down-regulating the expression of regulatory gene *Tri6* in T-2 toxin biosynthesis, thereby inhibiting T-2 toxin production [43]. As C8 oxylipins, 1-octen-3-ol treatment enhanced patulin production of *P. expansum* cultured on patulin-suppressing medium and up-regulated gene expression of patulin biosynthesis [7]. However, little studies have explored the signaling pathways or mechanisms of C8 MVOCs affecting patulin production. It has been reported that G protein signaling pathways positively regulated patulin production by modulating gene expression of patulin biosynthesis in *P. expansum* [20]. Therefore, we hypothesized that PePpo proteins affect patulin production in *P. expansum* by regulating C8 MVOC-mediated G protein signaling transduction.

## 5. Conclusions

*ppoA* deletion decreased C8 MVOC production in *P. expansum*, while they were not detected in the *ppoC* knockout strain. The deletion of *ppo* genes down-regulated gene expression involved in G protein-dependent cAMP-PKA and PLC/PKC signaling pathways. Moreover, the reduced growth and down-regulated expression levels of sporulation showed in the two mutants with ruffled and broken morphology of spores and hyphae. In addition, the two mutants had less pathogenicity on apple fruit and down-regulated patulin biosynthesis in vitro and in vivo. Notably, compared with the *ΔppoA* strain, the deletion of *ppoC* had more effects on the C8 MVOC production, fungal growth and development, pathogenicity and patulin production of *P. expansum*. However, the specific G protein-coupled receptors should be identified to elucidate the related action of mechanism in the future.

## Data Availability

The original contributions presented in the study are included in the article and Supplementary Materials, further inquiries can be directed to the corresponding authors.

## Supplementary Materials

The following supporting information can be downloaded at: https://www.mdpi.com/article/10.3390/jof10120827/s1, Table S1: All primers used for gene knockout and complementation. Table S2: Primers used for Real-time PCR of genes. Figure S1: PCR verification of *ppoA* and *ppoC* knockout mutants and their corresponding complementary strains of *P. expansum*. A and B showed the identification of the positive transformants of knockout mutants; C and D showed the identification of the corresponding complementary strains. Figure S2: Expression level of *ppoA* and *ppoC* in the mutants.
