# Supplementary material for: Oxygenase Ppo-Regulated Moldy Volatiles Affect Growth, Pathogenicity and Patulin Biosynthesis of Penicillium expansum Through G Protein Signaling
Source: J Fungi (Basel). 2024 Nov 27;10(12):827. doi: 10.3390/jof10120827 (PMC11678520; doi:10.3390/jof10120827)
Supplement: Supplementary file 1 [file jof-10-00827-s001.zip › jof-3309247-supplementary.pdf]

Supplementary Materials

Oxygenase Ppo-Regulated Moldy Volatiles Affect Growth, Pathogenicity and Patulin Biosynthesis of *Penicillium expansum* Through G Protein Signaling

Table S1. All primers used for gene knockout and complementation.

Gene	Primer Sequences (5' - 3')	Length (bp)
ppoA-up	F: AGCTCGGTACCCGGGGATCCTCTAGAGGCGTTCCTCTTACCCAG<br>R: TTGCATGCCTGCAGGTCGACTCTAGACAGATCCATCAGCCTTCC	1000
ppoA-down	F: TTGCCTAACTCGGCGCGCCGAAGCTTTCATGGAAGGAGCGAGAC<br>R: GTAAAACGACGGCCAGTGCCAAGCTTAGAAAGGACGAGCAACGT	1100
ppoC-up	F: ACAGCTATGACCATGATTACGAATTCGTTCGTGCAAGTTAAAGCG<br>R: GATCCCCGGGTACCGAGCTCGAATTCTGGTCTTAGGCTCGGTGT	1104
ppoC-down	F: TTGCCTAACTCGGCGCGCCGAAGCTTATGGTCCATTTGAGCAGA<br>R: GTAAAACGACGGCCAGTGCCAAGCTTAGCGGGTAGAACGAGTGA	1101
ppoA-C	F: TTGCCTAACTCGGCGCGCCGAAGCTT GGTGCATTCA GCCGTACGTA<br>R: GTAAAACGACGGCCAGTGCCAAGCTT GGGTGGTTGA TAGAAGAGAA	2439
ppoC-C	F: TTGCCTAACTCGGCGCGCCGAAGCTT GTGGGTCTGC GACGATCAAC<br>R: GTAAAACGACGGCCAGTGCCAAGCTT TAGCCATACT GCCTATCGCA	3484

Table S2. Primers used for Real-time PCR of genes.

Gene	Primer Sequences (5' - 3')	Gene ID	Target function
ppoA	F: GGCCAACTTTTCTCCCAATG<br>R: GACAAGCGCTGGATCTCAACT	PEX2_008260	Psi-producing oxygenase A
ppoC	F: ACCAGTGATGGCGCCTACTT<br>R: ATATCGCGGAATCCCAAAGA	PEX2_077570	Psi-producing oxygenase C
AC	F: CTTGCACAAGGCATCCGATA<br>R: CAGCGCTCAGGTGGTTGAAT	PEX2_061920	Adenylate cyclase
PkaC	F: GCAAATCGAGCACACAAATGA<br>R: AAGTTCCCCACAACGTAACGA	PEX2_021600	cAMP-dependent protein
PkaR	F: CCGGCTTAAGAGTGCCGTTA<br>R: GAGCGTTGAGGACGGTCAA	PEX2_028480	cAMP/cGMP-dependent protein kinase
pkc1	F: TGTGAAGTGCTTCGCAGACAT<br>R: ACCAGTAGCCCGACCATTCTC	PEX2_068640	PKc protein Kinases,
pkc2	F: CAAGTCCGAGAAGCGTGTTTT<br>R: TTTGGAAACAAGCGTGCAAGT	PEX2_087150	Protein Kinase C
plc	F: GGCTGTCGGTGATGGAAATC<br>R: GTTGTGCAGAGACGACGTCAA	PEX2_061150	Phosphoinositide phospholipase C
abaA	F: ATCATTTCGACGGCCTCTTG<br>R: ATGCATGCTGGGACCATTG	PEX2_109770	Conidiophore development regulator abaA
brlA	F: ACTTCGGCGACTCCATTGC<br>R: TTACCGCAGGGCATGTTCAT	PEX2_076900	Conidiophore development regulator brlA
wetA	F: ATCACCGCAACCCTGGAGTA<br>R: CGGTGTCATGGAAAGCAAAA	PEX2_105580	Developmental regulatory protein wetA
vosA	F: TCCAGATCATTTGCGGATCA<br>R: GGTCGTGTCGTTCGTTTCAA	PEX2_008170	Velvet factor
patA	F: AAAGGCCGGTGCATTGATC<br>R: TTGGAGGCTTTGGTGAGCAT	PEX2_082810	Acetate transporter
patB	F: GCCAGGCTATGCGATTGAGT<br>R: GCTGGAACCCTGTCCATTGT	PEX2_082800	Carboxylesterase
patC	F: TCCACCTGCGAATATCCCTTA<br>R: CATCGCCAGTGCCATTTTC	PEX2_082790	MFS transporter
patD	F: ATGAGATTCGTCTGCGCAAAG<br>R: CTACCCAAGCGGGATGAGATT	PEX2_082780	Alcohol dehydrogenase
patE	F: CATTCTCATCGGGCCTGAGT	PEX2_082770	GMC oxidoreductase

	R: TCGAAGCTCTTCCGGACATG		
patF	F: GCGAGTGAATTCGGCCAAT<br>R: GTCCGACCCAAAGGATGAAG	PEX2_082760	Hypothetical protein
patG	F: CGGCCGTCTTGAAGGAAAT<br>R: CTTGCCGTAGCGGGTGAATA	PEX2_082750	Amidohydrolase
patH	F: CATTTATCGGCGGTGTTCTGA<br>R: GATCAACGCTTGCACGATAGC	PEX2_082740	m-Cresol methyl hydroxylase
patI	F: GCAAACTCATTCCGCAAGGA<br>R: TGGTTCTTGCCATCGATCAC	PEX2_082860	m-Hydroxybenzyl alcohol hydroxylase
patJ	F: CGCCAGACATACCGCCATA<br>R: TTTGGTCGATCGGGACTGTT	PEX2_082870	Hypothetical protein
patK	F: GACGCTGGGCTACTGGATTG<br>R: TCGTGCGTGAGGCCAGTAT	PEX2_082880	6-Methylsalicylic acid synthase
patL	F: GCAGGAGATCCGTTTCAGACA<br>R: CCACTGACCGACGGTTACAAC	PEX2_082850	C6 transcription activator
patM	F: ACCCACAGCTGCACATGGA<br>R: AGCGAGAAGAGGCGGAAGA	PEX2_082820	ABC transporter
patN	F: CGTTCGATGTCGCTAGCAAA<br>R: GGCGATAATCACGTCAATTCG	PEX2_082830	Isoepoxydon dehydrogenase
patO	F: TCGCCTCCTGGTGTGTATCTT<br>R: AAGCGTGCCCAGTCATTCAG	PEX2_082840	Isoamyl alcohol oxidase
tublin	F: CTCCAGCTCGAGCGTATGAAC<br>R: GGCTCCAAATCGACGAGAAC	PEX2_027410	An endogenous control

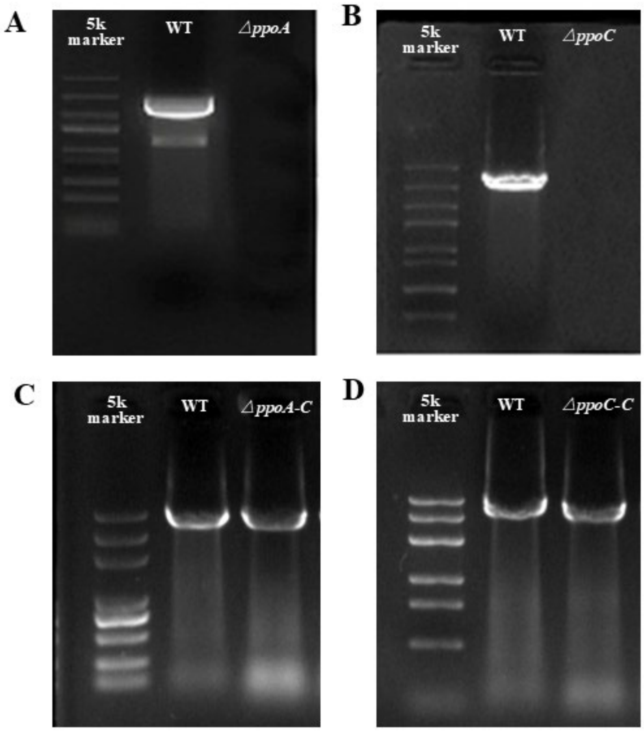

Figure S1. PCR verification of *ppoA* and *ppoC* knockout mutants and their corresponding complementary strains of *P. expansum*. A and B showed the identification of the positive transformants of knockout mutants; C and D showed the identification of the corresponding complementary strains.

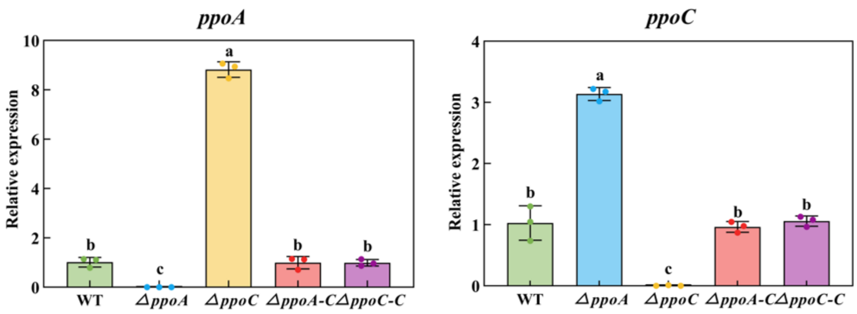

Figure S2. Expression level of *ppoA* and *ppoC* in the mutants.
